# Electrical level of defects in single-layer two-dimensional TiO_2_

**DOI:** 10.1038/srep15989

**Published:** 2015-11-02

**Authors:** X. F. Song, L. F. Hu, D. H. Li, L. Chen, Q. Q. Sun, P. Zhou, D. W. Zhang

**Affiliations:** 1 ASIC&System State Key Lab, School of Microelectronics, Fudan University, Shanghai 200433, China; 2 Department of Materials Science, Fudan University, Shanghai 200433, China; 3 Shanghai Engineering Research Center of Ultra-Precision Optical Manufacturing and Department of Optical Science and Engineering, Fudan University, Shanghai, 200433, China

## Abstract

The remarkable properties of graphene and transition metal dichalcogenides (TMDCs) have attracted increasing attention on two-dimensional materials, but the gate oxide, one of the key components of two-dimensional electronic devices, has rarely reported. We found the single-layer oxide can be used as the two dimensional gate oxide in 2D electronic structure, such as TiO_2_. However, the electrical performance is seriously influenced by the defects existing in the single-layer oxide. In this paper, a nondestructive and noncontact solution based on spectroscopic ellipsometry has been used to detect the defect states and energy level of single-layer TiO_2_ films. By fitting the Lorentz oscillator model, the results indicate the exact position of defect energy levels depends on the estimated band gap and the charge state of the point defects of TiO2.

Recently, many two-dimensional materials, such as graphene^1^,^2^ and TMDCs^3^,^4^, have received increasing attention due to their potential for applications in 2D nanoelectronic devices^5^,^6^. In fact, both the graphene and the TMDCs, used as channel materials in devices, are special materials of thin electrical and thermal conductor, with high carrier mobility properties. However, 2D nanoelectronic devices won’t be widely applied without the suitable 2D dielectric materials, which have rarely reported (except h-BN^2^,^7^). Due to the polar nature of the gate dielectrics used in MOSFETs, the carriers in the conducting channel couple electrostatically to the long-range polarization field created at the conductor/dielectric interface^13^. As a result, carrier mobility in two dimensional nanoelectronic devices is limited by surface optical phonons^13^,^14^, also limited by scattering from charged surface states and impurities^8^,^9^,^14^ and substrate surface roughness^10^,^11^,^12^. Even hexagonal boron nitride (h-BN) is an appealing substrate dielectric for use in improved 2D nanoelectronic devices, because it has an atomically smooth surface that is relatively free of dangling bonds and charge traps^7^, but h-BN is not suitable for being used in semiconductor manufacturing process, for it cannot be widely used in the CMOS manufacturing technology. As a potential 2D gate dielectric materials, single-layer TiO_2_ films can be act as the gate oxide in the 2D electronic devices.

With ~0.7 nm monolayer thickness^15^, single layer TiO_2_ is considered to be composed entirely of surface atoms, which endows its many special properties different from bulk titania, such as high crystallinity, wide bandgap of ~3.84 eV^16^, high chemical and thermal stability and large refractive index value, thus it involves certain useful applications, such as photocatalysts^17^, semiconductors^18^,^19^ and dielectric materials^20^. When the single-layer TiO_2_ films act as the gate dielectric layer, the electronic performance can be seriously affected by various defects, and the leakage current will occur in the oxide gate dielectric layer due to the electron trapping effect. In order to understand the influence of defect disorder on the electronic properties, it is essential to clarify the energetic position of the various defects inside the band gap, which is not yet available.

In general, deep-level transient spectra (DLTS) and thermally stimulated current (TSC) methods are used to measure the electrical levels of film defects. However, these methods have some limitations and special requirements for samples, hence they are difficult for this application. In this article, a nondestructive and noncontact method^21^ based on spectroscopic ellipsometry was introduced to investigate the optical properties and electrical levels of point defects of single-layer TiO_2_. Samples were fabricated by immersing the substrates in a colloidal suspension of single-layer TiO_2_ films and investigated by atomic force microscopy (AFM), X-ray photoelectron spectroscopy (XPS), and spectroscopic ellipsometry (SE). Based on the detailed SE analysis and fitted by the Lorentz oscillator model, we got the electrical levels of various different charged defects in single-layer TiO_2_ films.

## Results

**Figure 1:**
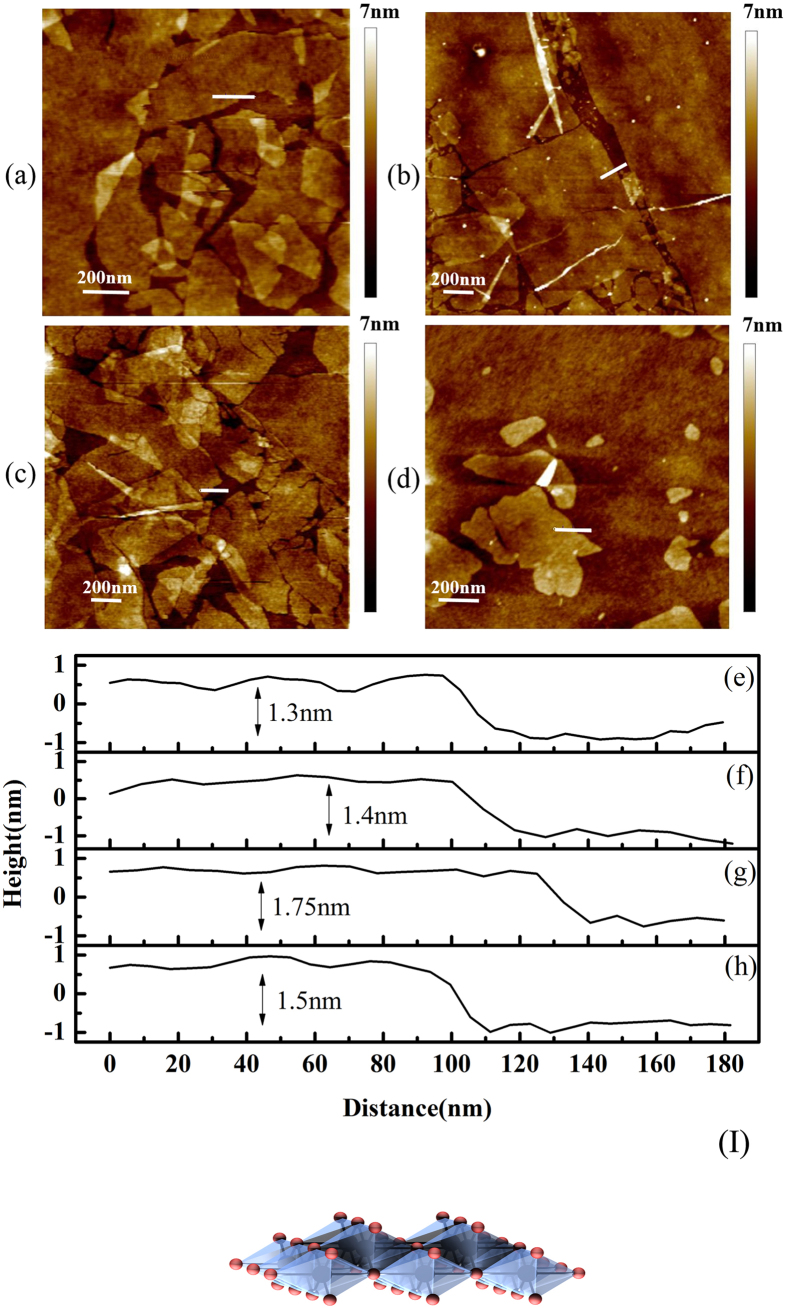
AFM analysis of single-layer TiO_2_ samples. The top column depicts the surface morphology of different samples: immersed in a colloidal suspension of single-layer TiO_2_ for 20 minutes for (**a**) sample 1, (**b**) sample 2, and (**c**) sample 3 and (**d**) immersed in colloidal suspension having been diluted 100 times for 30 seconds. The bottom is the height profile of marked line in (**a–d**), respectively, and structure model of single-layer Ti_1-δ_ O_2_ in (I).

The AFM results are shown in Fig. 1. After being immersed in a colloidal suspension of single-layer TiO_2_ films for 20 minutes, a few packed single-layer TiO_2_ films with non-ignorable gaps have been deposited on SiO_2_/Si substrate. The surface morphology of these samples is depicted in Fig. 1(a–c), while based on results from Fig. 1(e–g), the edge thickness of these samples ranges from 1.3 to 1.75 nm. In addition, the crystal structure model of the single-layer TiO_2_ was shown in Fig. 1(i). Ti atom is coordinated with six oxygen atoms and resulting TiO_6_ octahedra are joined via edge-sharing to produce the 2D lattice^22^.

**Figure 2:**
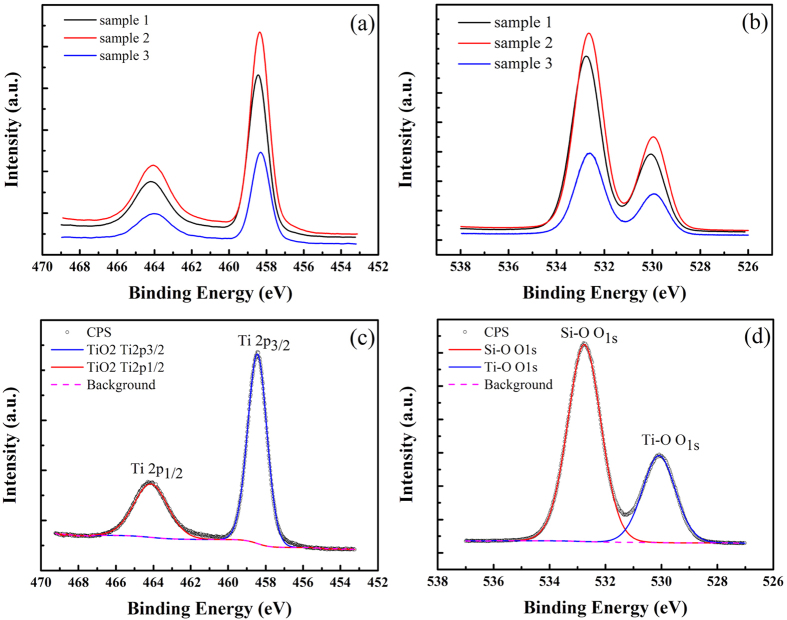
XPS spectra of (a) Ti 2p and (b) O 1s acquired on three samples immersed for 20 minutes, and fitted curves of (c) Ti 2pand (d) O1s peaks of sample 1.

Quantitative XPS analysis was measured on single-layer TiO_2_ samples to characterize the chemical state of the samples. As shown in Fig. 2(a,b), the spectra of Ti 2p and O 1s are observed at binding energies of 456–467 and 528–535 eV, respectively. From Fig. 2(c), the Ti 2p spectra of sample 1 is fitted with the Ti 2p_1/2_ and Ti 2p_3/2_ spin-orbital splitting photoelectrons peaks (area ratio is 2), located at binding energies of 464.03 and 458.35 eV, respectively. The FWHM of the Ti 2p_3/2_ signal was 1.206 eV for sample 1. Also the O 1s signal in Fig. 2(d) is fitted with two peaks: O 1s peak of Si-O species at 532.75 eV and O 1s peak of Ti-O species at 530.08 eV, closely resembling the reported values^23^,^24^. The values of the FWHM of the peaks were 1.409 eV and 1.391 eV, respectively.

According to XPS results, even the binding energy of Ti 2p peaks closely resemble the reported literature spectra^25^,^26^,^27^, and the peak separation of 5.68 eV between the Ti 2p_1/2_ and Ti 2p_3/2_ signals agree well with the reported values^28^, the binding energy of Ti 2p peaks still has a slight chemical shift to lower binding energy^28^. Furthermore, the ratio of titanium to oxygen in single-layer TiO_2_ samples, determined by integrating the areas under the Ti 2p and O 1s peaks and correcting the areas by the respective Scofield photoionization cross sections of the core level photoelectrons^29^, was about 0.748: 1. Therefore, we can deduce that the three single-layer samples exist oxygen vacancies.

**Figure 3:**
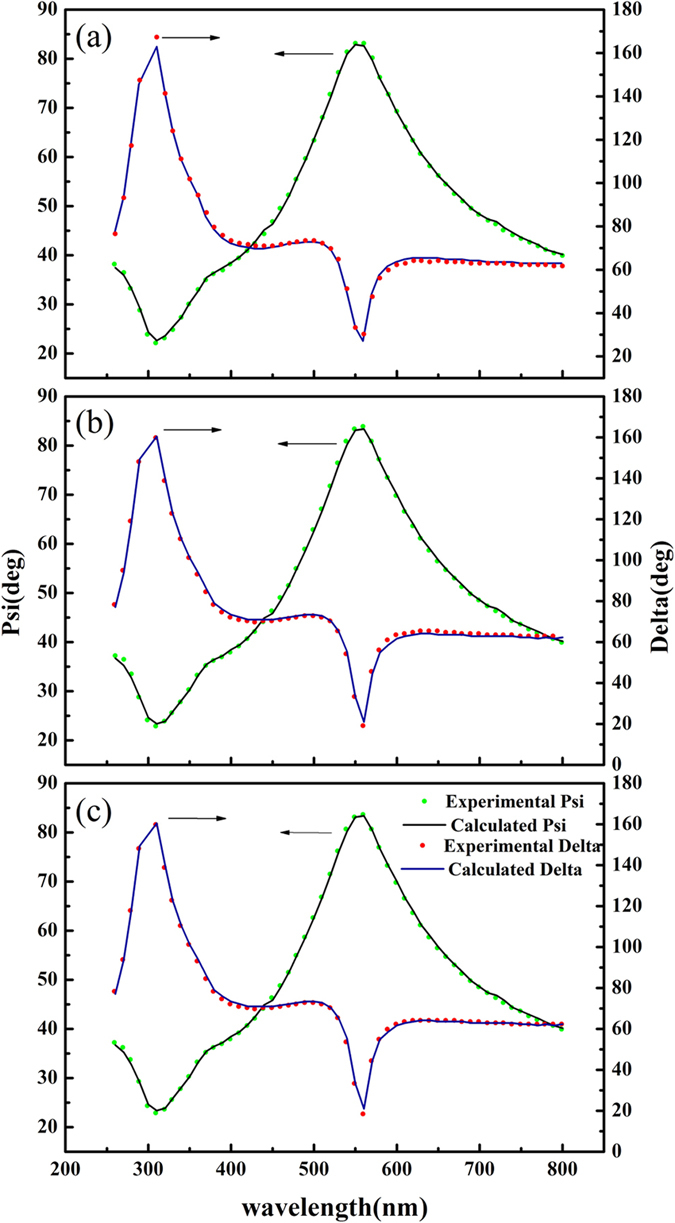
Experimental and calculated values for the ellipsometric parameters (a) Psi (Ψ) and (b) Delta (Δ) for single-layer TiO_2_ samples: (a) sample 1, (b) sample 2, and (c) sample 3 with incidence angle of 74°.

Figure 3 shows the spectroscopic ellipsometry results of the single-layer TiO_2_. To investigate the defect states and energy levels of single-layer TiO_2_ films, three Lorentz oscillators are used in the data analysis with key parameters listed in Table 1. Besides one oscillator used to describe the band-gap energy of single-layer TiO_2_ samples, two other oscillators in LOM were adopted to characterize two different charged defects.

From Fig. 3, the solid curves generated from the LOM dispersion obtained by fitting described above exhibited good agreement with experimental data. Also the thickness of three samples measured from atomic force microscopy were about 1.3, 1.4 and 1.75 nm, respectively, closely resembling the value of SE fitting results. Therefore, the three-oscillator model is suitable to characterize the single layer of TiO_2_ with different defects. However, the calculated results presented the value of A_3_ is the largest of A_1_–A_3_, indicating C_3_ is dominant oscillator of C_1_–C_3_ for three samples, and the oscillator center energies C_i_ for samples converge to three average values, 1.85 eV, 2.22 eV and 4.05 eV.

**Figure 4:**
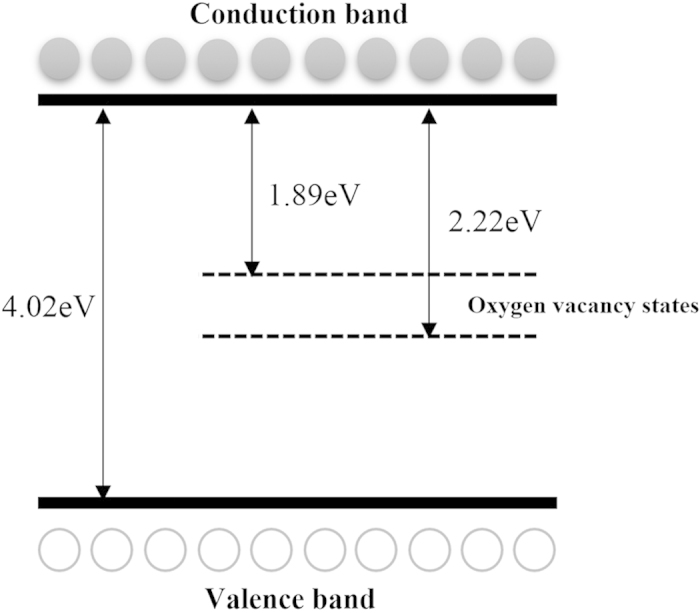
A band structure model for single-layer TiO_2_ with oxygen vacancies.

According to the definition of the LOM, these oscillators are fundamental characteristics of single-layer TiO_2_ with defects. While the probability of the electronic transitions from the conduction band to valance band or defect traps was expressed by the parameter A_i_, whose value represents the percentage contribution of oscillator i in the whole system. According to Table 1, the value of A_3_ is the largest of A_1_–A_3_, and the oscillator 3 center energy of 4.02 eV is very close to the bandgap energy of single-layer TiO_2_ (3.8 eV^30^), much larger than that of anatase TiO_2_ (3.2 eV), resulting in size quantization effects. Hence, the calculated band-gap energy for three single-layer TiO_2_ samples should be about 4.02 eV. Therefore, the remaining two oscillators 1 and 3 are used to characterize the two different charged defects appearance in the single-layer TiO_2_ samples. Combined with the XPS results, the center energies of C_1_ (1.89 eV) and C_3_ (2.22 eV) can be explained as two different oxygen vacancy defects. As shown in Fig. 4, the band structure of titania ultrathin films assembled by single-layer TiO_2_ films is obtained. In addition, the value of A_1_ is notably larger than A_2_, indicating the center energy located at 1.89 eV is the dominant defect configuration.

## Discussion

Figure 1(a–c) show the surface morphology of those samples which were immersed in colloidal suspension of single-layer TiO_2_ films for 20 minutes. From Fig. 1(e–g), the edge thickness of these samples ranges from 1.3 to 1.75 nm. However, the crystallographic height of single-layer TiO_2_ (0.7 nm) consist of the vertical distance between the levels of upper and bottom oxygen atoms of the host layer (0.42 nm) and the ionic radius of these two oxygen atoms (0.28 nm)^31^. There is big difference between the experimental thickness and crystallographic height. In order to ensure the ultrathin films assembled by single-layer TiO_2_, one substrate had been immersed in colloidal suspension having been diluted 100 times for 30 seconds. Then this sample was investigated by AFM, as shown in Fig. 1(d). While Fig. 1(h) shows that the edge thickness of the single-layer TiO_2_ films was ~1.5 nm. As a result, those samples shown in Fig. 1(a–c) were assembled by single-layer TiO_2_ films. However, the difference between the experimental thickness and crystallographic height is mainly caused by adsorbed charge-compensating protons, oxonium ions, or water molecules, as is the case for other nanosheets^19^,^32^,^33^,^34^,^35^.

In summary, we successfully deposited ~1.5 nm single-layer TiO_2_ on substrate and the properties of single layer TiO_2_ films have been investigated by AFM, XPS, and SE. The results of XPS demonstrate that some oxygen vacancy defects were formed in single-layer TiO_2_ samples. Also the parameters extracted from SE data by Lorentz oscillator model fitting illustrate that the probabilities and transition energies for different charged oxygen vacancy defects. Furthermore, the thickness of samples measured by atomic force microscopy exhibits excellent agreement with spectroscopic ellipsometry fitting values. Therefore, the method based on spectroscopic ellipsometry investigating the optical properties and electrical levels of point defects of single-layer TiO_2_ is appropriate. By fitting the Lorentz oscillator model, we get the electrical levels of different charged defects in single-layer of TiO_2_.

## Methods

### Fabrication of colloidal suspension of single-layer TiO_2_

According to the previous reported method^37^, reagents such as TiO_2_, K_2_CO_3_, Li_2_CO_3_ and MoO_3_ were mixed with a molar ratio of 1.73: 1.67: 0.13: 1.27. Then, the mixture was placed in a Pt crucible and reacted at 1473 K. After keeping this temperature for 10 h, the mixture was cooled spontaneously when the temperature reached 1223 K. By dissolving a K_2_MoO_4_ flux melt with water, the titanate crystals of K_0.8_[Ti_1.73_Li_0.27_]O_4_ were recovered and converted into a protonic form. After stirred in a 0.5 mol dm^−3^ HCl solution (2 dm^3^) at room temperature for 5 days, the acid-exchanged crystals, H_1.07_Ti_1.73_O_4_·H_2_O, were collected. Then, the protonic titanate crystals, H_1.07_Ti_1.73_O_4_·H_2_O, was attempted by reaction with a tetrabutylammonium hydroxide solution ((C_4_H_9_)_4_NOH; hereafter TBAOH) and little bit of protonic titanate was immersed in the TBAOH solution. After 10 days of vigorous shaking, colloidal suspension of single-layer TiO_2_ films was obtained.

### Fabrication and Measurement

**Figure 5:**
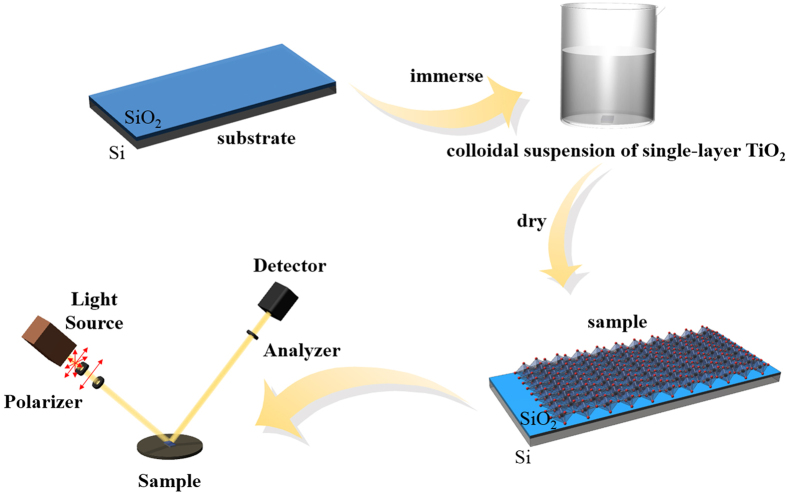
Fabrication of titania ultrathin films. SE was used to measure the ellipsometric parameters of single-layer TiO_2_ films on SiO_2_/Si wafers.

SiO_2_/Si wafers (2 × 2 cm^2^) were cleaned by ultrasonic treatment in acetone for 15 minutes, followed by ultrasonic treatment in absolute ethyl alcohol for 5 minutes. Before being immersed in a colloidal suspension of single-layer TiO_2_ films for 20 min, the SiO_2_/Si wafers were washed with copious water, as seen in Fig. 5. It should be noticed that the samples should be washed with water and dried before characterization. The surface topography and the thickness of three samples were measured under the ambient conditions by using a Veeco MultiMode VIII instrument equipped with a Nanoscope V controller. The XPS spectra of the samples were measured using ultra high resolution XPS analyzer PHOIBOS of SPECS customized UHV surface analysis system, while the SE spectra of the samples were measured using SOPRA GES5E Spectroscopic Ellipsometer range from 260 nm to 800 nm with a fixed incidence angle of 74°.

### Ellipsometry Modelling and Fitting

Ellipsometry is an optical technique used to investigate the dielectric properties of thin films that exploits phase information and the polarization state of light, and the physical thickness measurement can reach to angstrom resolution^36^. Also the Lorentz oscillator model (LOM) has been demonstrated that it is an effective way to fit the ellipsometric parameters tan Ψ and cos Δ, which are defined as


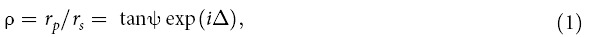


where *r*_*p*_ and *r*_*s*_ are the complex reflection coefficients of polarized light parallel and perpendicular to the incidence plane, respectively.

The SE spectra of the samples were analyzed by building a four-phase simple model structure consisting of substrate Si/SiO_2_/film (TiO_2_)/ambient. In this model, fitting variables include the unknown parameters of film thickness (d) and dielectric constant (ε). However, the LOM is suitable to characterize the dielectric function of the single-layer TiO_2_ films as follows:


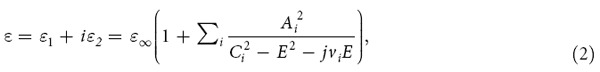


where ε_∞_ is the light-frequency dielectric constant; A_i_, C_i_, and *v*_*i*_ are the amplitude, center energy, and damping coefficient of each oscillator in eV, respectively. The A_i_ value also represents the percentage contribution of oscillator i in the whole system.

## Additional Information

**How to cite this article**: Song, X. F. *et al.* Electrical level of defects in single-layer two-dimensional TiO_2_. *Sci. Rep.* **5**, 15989; doi: 10.1038/srep15989 (2015).

## Figures and Tables

**Table 1:** Main parameters of the fitting results for three single-layer TiO_2_ samples; Amplitude A_i_ and center energy C_i_ have units of eV; d is the thickness of samples.

	Sample		
	1	2	3
A_1_	0.2344	0.2986	0.1732
C_1_	1.9985	1.7714	1.899
A_2_	5.70E-03	2.86E-03	3.10E-05
C_2_	2.2542	2.2139	2.2544
A_3_	2.7282	2.2577	0.3214
C_3_	4.0249	4.0553	4.0968
d (nm)	1.247	1.417	1.4846
